# Gender difference in oxidative stress: a new look at the mechanisms for cardiovascular diseases

**DOI:** 10.1111/jcmm.13038

**Published:** 2016-12-13

**Authors:** Melissa Christine Kander, Yuqi Cui, Zhenguo Liu

**Affiliations:** ^1^ Davis Heart & Lung Research Institute and Division of Cardiovascular MedicineThe Ohio State University Wexner Medical CenterColumbusOHUSA

**Keywords:** gender, oxidative stress, cardiovascular disease

## Abstract

Gender differences are present in many diseases and are especially prevalent in cardiovascular disease. Males tend to suffer from myocardial infarctions earlier than females, and a woman's risk of cardiovascular disease increases after menopause, suggesting a cardio‐protective role of estrogen. However, hormone replacement therapy did not decrease the risk of cardiovascular disease in post‐menopausal women; thus, other mechanisms may be involved besides estrogen. Oxidative stress plays an important role in the development of cardiovascular diseases such as coronary artery disease. Gender is also associated with differences in oxidative stress. Under physiological conditions, females appear to be less susceptible to oxidative stress. This may be due to the antioxidant properties of estrogen, gender differences in NADPH‐oxidase activity or other mechanism(s) yet to be defined. This review strives to discuss gender differences in general terms followed by a more detailed examination of gender differences with oxidative stress and various associated diseases and the possible mechanisms underlying these differences.

## Introduction

Differences between males and females can be seen in many diseases such as coronary artery disease (CAD). The prevalence, presentation, severity and outcome for a variety of diseases have been known to vary based on gender. One example is Alzheimer's disease, which disproportionally affects women more than men in both prevalence and severity 1. Multiple sclerosis (MS) also has a higher prevalence in women than in men, and the female‐to‐male ratio of patients with MS has been increasing over the past several decades 2. However, not all diseases affect women more than men like Alzheimer's disease, and MS. Parkinson's disease presents with a more benign phenotype in women and the incidence of the disease is also lower in women 3. Besides these medical conditions mentioned here, there are many other diseases displaying gender differences. These differences are important because it is possible that different therapies and treatments could be targeted for men and women.

Cardiovascular diseases are one of the areas in particular where gender differences have been studied extensively. In this review, the general gender differences in cardiovascular diseases will be discussed. Then, more specifically, gender differences in oxidative stress will be examined. Oxidative stress is considered an important mechanism for the development of cardiovascular diseases especially atherosclerosis 4, 5. Because gender differences in cardiovascular diseases are prevalent, it is important to study the link between gender differences in oxidative stress, which will be the focus of this review, although oxidative stress associated with other disease states will also be discussed briefly.

## Gender differences in cardiovascular diseases

Gender differences have been observed and studied in a wide range of cardiovascular diseases, including myocardial infarctions (MI), heart failure, hypertension, shock, cardiac hypertrophy, as well as other diseases involving the cardiovascular system as summarized in Table 1. Some of these differences have been so pronounced in animal studies that researchers have suggested that at some level, male and female hearts are functionally distinct from one another 6. These differences stem from the observation that pre‐menopausal women are relatively protected from cardiovascular diseases when compared to men possibly due to cardio‐protective effects of estrogen 6. The role of estrogen will be discussed more extensively in a later section of the review.

**Table 1:** Gender differences in cardiovascular diseases and risk factors

Disease or Risk factor	Females	Males
Myocardial infarction	10 years older than males with first MI, higher mortality in younger ages	Younger than females with first MI, but lower mortality
Heart failure	Lower incidence, diastolic heart failure more common	Higher incidence, systolic heart failure more common
Hypertension	Lower incidence in younger females	Develop earlier and more severe hypertension
Cardiac hypertrophy	Decreased	Increased
Ischaemia–Reperfusion injury	Decreased	Increased
Diabetes	Higher increased risk of CD	Lower increased risk of CD
HDL levels	Higher levels	Lower levels
Triglyceride levels	Higher increased risk of CD	Lower increased risk of CD
Total cholesterol	Levels rise in menopausal transition period	Levels lower than post‐menopausal females
LDL levels	Levels rise in menopausal transition period	Levels lower than post‐menopausal females

Differences between males and females in different cardiovascular diseases and cardiovascular risk factors including diabetes, HDL, LDL, triglyceride and total cholesterol levels were summarized.

CD: cardiovascular diseases; HDL: high‐density lipoprotein; LDL: low‐density lipoprotein.

Gender differences have been seen in MI. Typically women are older than men when they have their first MI 6. In fact, women who present with a ST‐elevation MI (STEMI) are almost 10 years older than men 7. Although often older than men, the younger a woman is when she has her first MI, the greater her risk of death compared to an age‐matched male 6. One study with younger patients of 30–54 years of age showed that women represented 25% of hospitalizations for acute MI yet these patients had higher incidence of in‐hospital mortality compared to males 8. These data indicate that pre‐menopausal women are less likely to have a MI compared to males of the same age, but are more likely to suffer from comorbidities or more severe MI resulting in a higher mortality. The high mortality rate in females did decrease significantly over the 10‐year period, while the mortality rate in males did not change significantly 8. In addition to the age of presentation and severity, women are more likely than men to present with atypical MI symptoms. Women are more likely than men to present without chest pain 9. Women are also less likely to have anatomical obstructive CAD than men, but are more likely to present with myocardial ischaemia and thus ischaemic heart disease 10.

Gender variation also can be seen in patients with heart failure. The incidence of heart failure is higher in men than in women 6. The prevalence of heart failure is about the same for both males and females 6. This is because even though more males are diagnosed with heart failure, the number of males and females living with heart failure is similar as women with heart failure usually have a lower mortality rate than men. There is also a difference in the most common type of heart failure in men and women: diastolic heart failure occurs more often in women, whereas systolic heart failure occurs more often in men 6.

**Figure 1:**
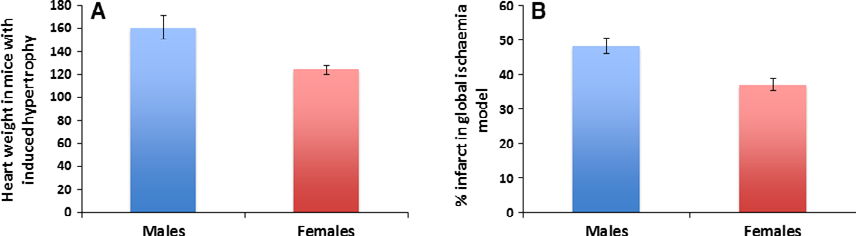
Male and female differences in hypertrophy and ischaemia–reperfusion injury. (**A**) In an animal study with mice, females were shown to have lower levels of hypertrophy than males. Figure adapted from Skavdahl *et al*. 16. (**B**) In a global ischaemia model of rat hearts, females were shown to have a smaller percentage of infarct than males. Figure adapted from Bae and Zhang 17.

Hypertension, cardiac hypertrophy and ischaemia–reperfusion injury are three other major cardiovascular conditions where gender differences are present. Young adult women are less likely to develop hypertension than men 11. Also, animal studies have shown that males develop an earlier and more severe hypertension than females 12, 13, 14. Males are also shown to have increased cardiac hypertrophy compared to females 15, 16 (Fig. 1A). Females are also more likely to have relatively preserved left ventricular function 10. In addition to reduced hypertrophy, studies have also shown that females have reduced ischaemia–reperfusion injury 15, 17 (Fig. 1B). The beneficial outcome in ischaemia–reperfusion injury in females may be due to the effect of estrogen. Elevated calcium levels increase ischaemia–reperfusion injury, and estrogen may lower the calcium levels before ischaemia leading to less injury in females 15.

**Figure 2:**
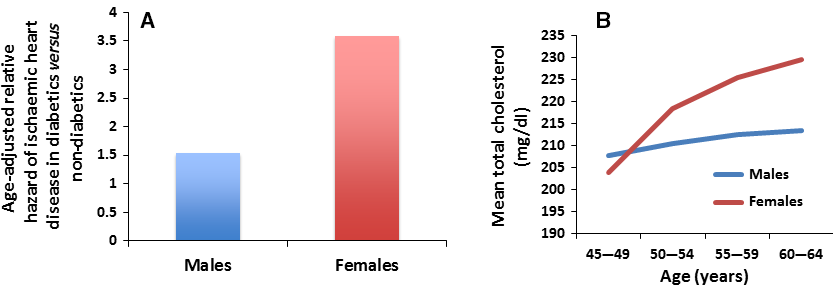
Cardiovascular risk factors and gender. (**A**) Diabetic females have an increased risk of developing coronary artery disease compared to diabetic males. Both diabetic males and females have an increased risk of cardiovascular disease compared to non‐diabetics. This figure was modified from Goldschmid *et al*. 19. (**B**) Pre‐menopausal women have lower levels of total cholesterol than men. Levels in post‐menopausal women though rise and exceed that of age‐matched men. Figure modified from Brown *et al*. 20.

In addition to cardiovascular diseases themselves, there is also a link between several cardiovascular disease risk factors and gender. Of the many cardiovascular disease risk factors, diabetes, high‐density lipoprotein (HDL) levels and triglyceride levels have more of an impact on cardiovascular disease risk in women than in men 18. Diabetic men for example have a two‐ to threefold increased risk of developing CAD, whereas a diabetic woman has a three‐ to sevenfold increased risk 18, 19 (Fig. 2A). Thus, diabetic women have an even higher risk of developing heart disease compared to diabetic men. High‐density lipoprotein levels are higher in women from young adulthood onward although there is some discrepancy where some studies describe a decrease in HDL levels after menopause 18. Higher HDL levels in female would lead to a lower risk of cardiovascular diseases. While elevated triglyceride levels are a risk factor for both men and women, elevated triglycerides levels increase the CAD risk more in women than in men 18. There are also differences in risk factors before and after menopause, suggesting a possible role of estrogen. Total cholesterol and low‐density lipoprotein (LDL) levels in post‐menopausal women rise and exceed the values in men 18, 20 (Fig. 2B). Estrogen acts as a LDL receptor up‐regulator; thus, the increase in LDL levels after menopause could be likely due to decreased estrogen levels. These changes in LDL and total cholesterol levels may actually begin prior to menopause in the perimenopausal period. One study has shown that increases in total cholesterol and LDL levels are significant around the time of the last menstrual period and these increases are greater than the changes seen before or after the last menstrual period 21. The menopausal transition therefore may be a crucial time when lipid levels increase in females prior to the actual onset of menopause.

## Estrogen and cardiovascular disease

As discussed previously, ischaemia–reperfusion injury and LDL levels may be attenuated by the presence of estrogen in pre‐menopausal women. The question then remains whether the presence of estrogen could explain the gender differences in various cardiovascular diseases or whether other mechanisms are involved. This section will briefly discuss several studies looking at the possible cardio‐protective properties of estrogen.

Sex hormones including estrogen are present globally with their receptors in the heart 6, suggesting that the sex hormones may exert their effects on cardiovascular system by affecting the heart itself. In animal models, estrogen has been shown to improve heart function and decrease the severity of injuries 6. Estrogen may also act as an antioxidant, which could contribute to its cardio‐protective properties. These data along with the fact of significantly lower mortality in pre‐menopausal women led to the belief that estrogen could act as a cardio‐protective agent 22. Several studies, including the HERS trials and the Women's Health Initiative study 23, 24, were designed to test this hypothesis by determining whether hormone replacement therapies (HRT) could decrease the incidence or severity of cardiovascular diseases in post‐menopausal women. The HERS I/HERS II trials demonstrated no cardiovascular benefit of HRT during the 6.8 years of follow‐up 23. In addition, there might have been an increased CAD risk during the first year of HRT, and there was an increased risk of non‐fatal ventricular arrhythmias among the women on HRT 23. The Women's Health Initiative study showed that there was an increased cardiovascular risk for post‐menopausal women taking the estrogen–progestin hormone combination 24. A comprehensive systematic review revealed that HRT had no cardiovascular benefit and that estrogen therapy alone significantly increased the risk of stroke when compared to placebo 25. The results of these HRT trials suggested that estrogen was not cardio‐protective in post‐menopausal women and might actually be more harmful than helpful from a cardiovascular standpoint. Therefore, the role of estrogen and its effect on the cardiovascular system are still unclear. Estrogen may explain some of the gender differences in cardiovascular diseases especially in pre‐menopausal women, but it is not the only mechanism (at best) at play. Further studies are needed to identify other mechanism(s).

## Oxidative stress

Oxidative stress is a condition that occurs when the rate of reactive oxygen species (ROS) formation exceeds the rate of the antioxidant defence system 26. Reactive oxygen species is generated as natural by‐products of normal oxygen metabolism and has important roles in cell signalling and homoeostasis in normal conditions 27. However, at times during disease states (such as inflammation or infection) and environmental stress (*e.g*. UV or heat exposure) or ionizing radiation, ROS levels could increase dramatically and may result in significant damage to cellular structures 27. Oxidative stress is considered an important mechanism for the development of cardiovascular diseases 4, 5. Hyperlipidaemia and diabetes in particular are both associated with increased oxidative stress, which may lead to the development of atherosclerosis 4, 5. In addition to cardiovascular diseases, other diseases and ageing are accompanied with oxidative protein damage 28. Because oxidative stress plays such an important role in various diseases, it is important to study any gender differences associated with oxidative stress.

Reactive oxygen species are formed normally in most cells and play an important role in cell function and inflammatory response including cell‐fate signalling, cell proliferation, gene transcription and gene expression 29, 30. In addition to its physiological function, ROS also play a pathologic role in oxidative stress‐induced cell injury and death 29, 30. There are several types of ROS produced in the body including free radicals superoxide and hydroxyl, and non‐free radicals such as hydrogen peroxide 31. Hydrogen peroxide is a relatively stable ROS that is formed as a product of the free radical superoxide by the enzyme superoxide dismutase (SOD) 32. The production of hydrogen peroxide is dramatically increased during the oxidative burst in inflammatory conditions, including the response to lipopolysaccharides both *in vitro* and *in vivo* 33, 34, 35. There are many sources of ROS, but NADPH‐oxidases are important sources of ROS within the vasculature 36. NADPH‐oxidases generate superoxide radicals by transferring electrons from NADPH to molecular oxygen through a ‘Nox’ subunit 36. In addition to ROS, a number of reactive nitrogen species including nitric oxide and peroxynitrite are also produced in vascular cells 31.

**Figure 3:**
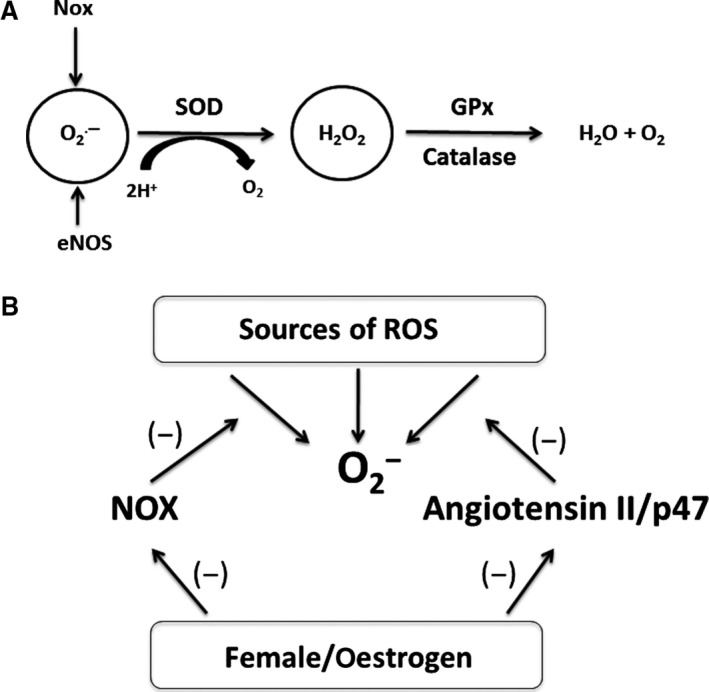
(**A**) Antioxidant Enzymes. NADPH‐oxidase (‘Nox’ subunits) and eNOS both contribute to the production of superoxide. Superoxide is broken down to hydrogen peroxide by superoxide dismutase (SOD). Hydrogen peroxide is metabolized to water and oxygen by either catalase or GPx. Any imbalance in this anti‐oxidant system could lead to increases in oxidative stress. (**B**) Mechanisms of Gender Differences in NADPH‐Oxidase**.** Normally, angiotensin II binds to the angiotensin type 1 receptor (AT1), ultimately resulting in p47 phosphorylation. P47 then travels to the plasma membrane participating in the assembly of the NADPH‐oxidase complex, allowing for superoxide production. Females have lower levels of p47 both in the cytoplasm and at the cell membrane, leading to lower levels of superoxide production (not dependent on estrogen). Females also have lower levels of NADPH‐oxidase activity (may be estrogen‐dependent, depicted as the Nox subunit). The ultimate result of these gender differences is lower levels of superoxide in females with lower levels of oxidative stress and decreased cardiovascular disease risk.

Under normal conditions, cells are protected from ROS by antioxidant defence mechanisms including enzymes such as SOD, catalase and glutathione peroxidase (GPx) 26. As mentioned previously, SOD rapidly catalyses the conversion of superoxide to hydrogen peroxide. Three forms of SOD exist including CuZn‐containing SOD (SOD1), Mn‐containing mitochondrial SOD (SOD2) and CuZn‐containing extracellular SOD (SOD3) 31. Hydrogen peroxide is metabolized to oxygen and water by either catalase or GPx 31, 32 (Fig. 3A). These antioxidant enzymes are crucial for the normal redox balance in cells. When this redox balance is interrupted, and more ROS are present than the antioxidant defence system can handle, oxidative stress and cell damage result.

## Gender and oxidative stress

As discussed previously, the association between gender and oxidative stress is important because oxidative stress is implicated in many diseases that present differently in males and females. This section of the review will focus on the relationship between gender and oxidative stress (Table 2). It was reported that oxidative stress was higher in male rats than female rats 37. Another study showed that *in vivo* biomarkers of oxidative stress were higher in young men than in women of the same age 38. Similarly, it was observed that ROS production was higher in the vascular cells from males than in the cells from females 39. In addition, clinical and experimental data suggested a greater antioxidant potential in females over males 40. These studies indicate that there is an apparent association between gender and oxidative stress, where women seem to be less susceptible to oxidative stress.

**Table 2:** Gender differences in oxidative stress

Females	Males	Literature Source
Lower oxidative stress level	Higher oxidative stress level	37
Lower oxidative stress biomarkers	Higher oxidative stress biomarkers	38
Lower ROS production	Higher ROS production	39
Greater antioxidant potential	Lower antioxidant potential	40

Females generally have lower levels of oxidative stress and ROS production than males.

ROS: reactive oxygen species.

For oxidative stress to occur, there needs to be an imbalance between ROS production and the antioxidant defence system. There appears to be a difference in the expression and/or activities of antioxidant enzymes between males and females (Table 3). These enzymes are present in multiple tissues in the body. In regard to SOD, there is no uniform consensus on gender differences, but it has been suggested that there may be a difference in certain tissues. It was reported that brain and lung SOD activity levels were higher in female mice, but there was no significant difference in SOD activity levels between male and female mice in the kidney or heart 41. In another study, female rats were found to have higher SOD activity levels in the heart than the males 37. Interestingly, the SOD activity levels in both male and female rats were significantly decreased after castration compared to their respective controls 37, suggesting that there could be an association between sex hormones and SOD activity levels. However, some studies showed no difference in SOD activity levels between males and females; thus, some discrepancy regarding the association of SOD activity and gender still remains 38, 42. Catalase activity levels were found to be the same between genders in the brain, heart, lung and heart, but higher in the female kidney 41. However, some studies showed no difference in catalase activity levels between males and females 37, 38, 43. These data suggest that catalase activity and thus hydrogen peroxide degradation may not be affected by gender and sex hormones. Several studies demonstrated that GPx activities were lower in females compared to males 37, 38, 41. After castration, there was no significant change in GPx activity levels, suggesting that GPx might not be influenced by sex hormones 37. Glutathione peroxidase was the only antioxidant enzyme that consistently showed a gender bias across several studies. The fact that GPx levels were lower in females seemed counterintuitive, as females were believed to be less susceptible to oxidative stress than males. These observations suggested that other mechanism(s) had to be present in females to overcome the lower levels of GPx.

**Table 3:** Gender differences in enzyme expression

Enzyme	Females	Males	Literature source
SOD	No uniform consensus but suggested that activity levels higher in brain, lung, and heart	No uniform consensus but suggested that activity levels lower in brain, lung, and heart	37, 38, 41, 42
Catalase	No difference	No difference	37, 38, 41, 43
GP_x_	Lower levels not affected by castration	Higher levels not affected by castration	37, 38, 41
Nox1	Lower expression	Higher expression	36, 45
Nox2	No uniform consensus but three studies showing no difference and one showing lower expression	No uniform consensus but three studies showing no difference and one showing higher expression	36, 44, 45, 46
Nox4	No uniform consensus with two studies showing lower expression and one showing higher expression	No uniform consensus with two studies showing higher expression and one showing lower expression	36, 44, 45

Of the anti‐oxidant enzymes, catalase showed no gender differences whereas glutathione peroxidase (GP_x_) levels were lower in females than in males. SOD expression varied between studies with some showing no difference and some showing higher levels in females than in males in the brain, lung and heart. Two subunits of NADPH‐oxidase were higher in males than in females.

In addition, NADPH‐oxidase subunits were found to exhibit gender discrepancies. Expression of Nox1 and Nox4 was higher in males than in females, suggesting that gender differences in superoxide formation could be due to the activity of these two subunits 36. This was consistent with a recent finding that Nox4 levels were significantly lower in the mesenteric arteries of female rats compared to the males 44. The above two studies were also consistent in showing that Nox2 levels did not differ between males and females 36, 44. Another study using pigs showed higher levels of Nox1 and Nox2 in porcine isolated coronary arteries (PCA) in males although Nox4 was higher in females 45. It could be possible that higher expression of Nox subunits in men could in part explain why males exhibit higher levels of oxidative stress than females (Fig. 3B). Wong *et al*. 45 also showed that the Nox subunits played a role in endothelium‐derived hyperpolarization in PCAs of males but not females, which could further show that increased level of Nox activity is indicative of more oxidative stress in males.

At the molecular level, there have been a few studies on the differences between males and females in regard to oxidative stress and gene and protein expressions. As mentioned above, Nox1 and Nox4 expression was found to be higher in males in the basilar artery 36. This same study found that protein expression of Nox2, SOD1, SOD2 and SOD3 did not differ between genders 36. These data were consistent with another study, which showed that protein expression of Nox2, endothelial nitric oxide synthase (eNOS) and SOD1‐3 was similar in males and females 46. There are few studies on gender differences in gene expression, and further research is needed. There was one study that showed the mRNA expression of SOD2 was similar in males and female rats 47. Another study showed that pre‐menopausal females with total hysterectomy and bilateral salpingo‐oophorectomy had a reduction in mRNA expression of SOD and GPx after surgery that recovered after ERT 48. Catalase mRNA expression was not changed by surgery 48. This suggests that gene expression of SOD and GPx is estrogen‐dependent, whereas gene expression of catalase is not. Further research is needed to better explore the role that gender plays in the gene expression of anti‐oxidants and other genes associated with oxidative stress.

While there may be some differences in antioxidant enzyme activity levels between males and females as discussed previously, the greatest difference in antioxidant properties is likely due to estrogen. Estrogen acts as an antioxidant by scavenging free radicals due to the presence of a phenolic hydroxyl group 37. The antioxidant action of estrogen could explain the gender differences with GPx. Because estrogen acts as a potential antioxidant in females, less GPx is needed in females compared to males who would not benefit from the antioxidant properties of estrogen. Indeed, it was found that after castration, oxidative stress was higher in female rats compared to female controls, but there was no significant difference in males after castration 37. Estradiol has been shown to up‐regulate Mn‐SOD gene expression through activation of a MAP kinase signalling pathway, which is another example of the anti‐oxidant effects of estrogen 49. There have been some studies that showed no correlation between estrogen levels and oxidative stress 38. This does not mean that estrogen does not have antioxidant properties, but there are likely other mechanisms contributing to the lower oxidative stress in pre‐menopausal women. Other than being a potential antioxidant, estrogen has also been found to increase mitochondrial ROS production, which is involved in cell signalling pathways 50. Clearly, estrogen has a complex role, and it is possible that its effect on oxidative stress is multifaceted.

## Gender, oxidative stress and diseases

There are many diseases associated with oxidative stress. A clear difference in gender and oxidative stress could be observed in various diseases, including autoimmune thrombocytopenia (AITP), pancreatic disease, ageing, thyroid disease and some cardiovascular diseases such as CAD. Autoimmune thrombocytopenia is a bleeding disorder, and women with AITP have significantly higher levels of oxidative stress compared to both female controls and males with AITP 51. This suggests that there is a gender‐specific difference in the pathophysiology of AITP. In regard to pancreatic disease, female rats exhibit higher protection against oxidative stress and thus have a lower risk of developing insulin resistance compared to male rats 43. In addition to these disease states, oxidative stress and gender have been shown to be associated with ageing. The markers of oxidative protein damage are significantly higher in male rats than in female rats of the same age 28. The rate of ROS production by mitochondria in females is also significantly lower than that of males, which has been implicated in ageing 52, 53. Thyroid disease is more prevalent in females, and one study found that adult female thyroids produced significantly higher amounts of hydrogen peroxide than males 54. This same study found that Nox4 levels were 1.5‐fold higher in adult female thyroids than male thyroids 54. These observations show that the gender differences associated with oxidative stress in various disease processes could have important implications. Further studies are needed to determine whether gender plays an important role in other disease states associated with oxidative stress.

The most apparent association between oxidative stress and gender can be seen in the cardiovascular system where oxidative stress has a very prominent effect. Recent studies suggest that levels of vascular ROS may be lower in females than in males during physiological conditions 31. One such study found that under normal conditions, the male rat aortas generated more superoxide radicals than the female aortas 42. In contrast, a study comparing CAD in post‐menopausal women with males demonstrated that even in the control groups who did not have CAD, post‐menopausal women were found to have higher oxidative stress levels than men 55. This suggests the possibility that estrogen lowers oxidative stress in pre‐menopausal females. A study investigated the effects of NADPH‐oxidase activity on the cerebral circulation and found that superoxide production was significantly lower in the basilar and middle cerebral arteries of female rats 36. This same study, however, showed no gender difference in the vascular response to hydrogen peroxide, thus suggesting that the difference between male and female ROS production could be due to NADPH‐oxidase activity 36. As mentioned previously, the enzyme SOD rapidly converts superoxide to hydrogen peroxide. There was some discrepancy as to whether SOD activity levels differed between males and females, with several studies showing no difference. It is therefore less likely that the difference in superoxide levels is due to SOD activity. It is more likely that males could have higher levels of superoxide production due to NADPH‐oxidase activity.

Indeed, it has been shown that male rats have higher levels of Nox subunits as mentioned earlier in the review; thus, gender differences in NADPH‐oxidase activity could be due to these subunits. In addition, ovariectomy of female rats resulted in an increase in NADPH‐oxidase activity; treatment with 17β‐estradiol restored NADPH‐oxidase activity to normal levels 36. These data further suggest that estrogen may contribute to decreased oxidative stress, possibly *via* the regulation of NADPH‐oxidase activity especially the Nox1, Nox2 and Nox4 subunits. Estrogen therefore may contribute to the differences in superoxide levels between males and females.

It has also been suggested that males have higher NADPH‐oxidase activity levels due to an angiotensin II‐mediated mechanism 12, 40. A study on spontaneous hypertensive rats shows that females are less susceptible to angiotensin II‐mediated increases in oxidative stress 40. Another study with wild‐type mice had similar findings, showing that angiotensin II‐stimulated production of superoxide and hydrogen peroxide in cerebral arteries from females was approximately 75–85% lower than males 46. This same study also showed that superoxide production decreased in male Nox2 knockout mice, but not in the females, suggesting that Nox2 plays a role in mediating ROS generation in response to angiotensin II in males but not in females 46. Another study found that males have higher levels of p47, which is a cytoplasmic subunit of NADPH‐oxidase 56. This subunit is connected to the angiotensin II system as angiotensin II binds to the angiotensin type I receptor, which then induces the phosphorylation of p47. Phosphorylated p47 then translocates to the plasma membrane, which is an essential step in the assembly of the NADPH‐oxidase complex and the initiation of ROS production. This study found that p47 levels did not change with estrogen levels, thus representing an estrogen‐independent mechanism. These data suggest that regulation of NADPH‐oxidase activity may be more complex and likely the result of a combination of estrogen‐dependent and estrogen‐independent mechanisms (Fig. 3B).

In addition to physiological conditions, studies have been conducted to evaluate the relationship between oxidative stress and gender under pathologic conditions such as hypertension, diabetes, CAD and shock. Male spontaneously hypertensive rats were found to have higher levels of superoxide generation than female rats 12. As discussed above, it is likely that gender differences associated with NADPH‐oxidase activity are responsible for the higher levels of superoxide in males. Male spontaneous hypertensive rats also have lower levels of nitric oxide (NO) due to its degradation by superoxide, thus contributing to oxidative stress 12. The nitric oxide pathway can also be affected by ROS, which oxidize the eNOS cofactor tetrahydrobiopterin (BH_4_) which then uncouples eNOS resulting in an increased production of superoxide 57. This mechanism appears to be related to sex although more research is needed to determine the exact role sex plays 58. One study showed that mRNA expression of eNOS was increased in ovariectomized pigs compared to gonadally intact female and male pigs although there was no difference in the protein expression of eNOS 59. Another study with diabetic rats demonstrated that eNOS mRNA expression was higher in the aorta of normal female controls compared to male controls although the mRNA level was the same in males and females with diabetes 60. This same study found that Nox subunits also played a role in diabetes. Han *et al*. 60 found that female diabetic rats had significantly higher levels of Nox1 and Nox4 than controls and other groups. This may explain why the female aorta is predisposed to injury early in diabetes. These results were consistent with the findings that mRNA expression of Nox2 and Nox4 was higher in the mesenteric arteries of female diabetic rats 44, and could explain why diabetic females are at a higher risk of developing cardiovascular diseases.

A study on CAD in males and post‐menopausal females showed that women with CAD had oxidative stress levels that were almost three times that of men with CAD 55. Another study focusing on peripheral artery disease (PAD) demonstrated that African‐American women had higher levels of ROS than African‐American men, but there was no significant gender difference in Whites 61. This same study found that women with PAD had more pro‐inflammatory biomarkers than men with PAD 61. It is possible that lower amounts of estrogen in post‐menopausal females could explain this gender difference in PAD and CAD, although the results of the HERS and Women's Health Initiative trials suggest that the mechanism may be more complex. Further studies are needed to determine why post‐menopausal women with CAD exhibit more oxidative stress than their male counterparts.

In regard to shock, a study was conducted with lipopolysaccharides (LPS) to induce inflammation and oxidative stress to simulate shock 62. It was found that female rats treated with LPS had a greater stroke volume and cardiac output compared to male rats, while male rats with LPS treatment were found to have systolic dysfunction after 24 hrs whereas female rats did not 62. These data reveal a significant gender difference associated with LPS injection and that female rats seem to recover cardiac function faster than male rats. Overall, the results of all these studies have demonstrated that there is a clear association between gender, oxidative stress and various cardiovascular diseases. The question still remains whether these gender differences with oxidative stress explain the gender differences in the overarching diseases.

## Conclusion

Gender differences are present in many diseases, especially in cardiovascular diseases. It was postulated that estrogen could have cardio‐protective properties, thus explaining why pre‐menopausal women are less likely than men to suffer from heart diseases such as CAD. However, hormone replacement therapy had no cardiovascular benefits and might in fact cause an increased risk of cardiovascular diseases. The role of estrogen may not be as clear‐cut, and more research is needed to understand the reason behind these gender differences.

Oxidative stress is associated with a variety of diseases including diabetes mellitus, hypertension and atherosclerosis. Oxidative stress is therefore an important mechanism in cardiovascular diseases, and any gender differences associated with oxidative stress could have implications in the mechanisms for these cardiovascular diseases. Females prior to menopause appear to have lower levels of oxidative stress compared to men. One reason for this apparent gender difference could be due to the anti‐oxidant properties of estrogen. However, anti‐oxidant enzyme activity levels, NADPH‐oxidase level (especially p47, Nox levels) and angiotensin II may also play an important role. It is very likely that multiple mechanisms are responsible for the gender differences, and estrogen may not be the only reason for the differences between males and females. Further research is needed to determine the extent of the role of estrogen and other mechanisms that may be involved.

## Conflict of interest

The authors confirm that there are no conflict of interests.
